# Supplementary material for: Functionalized Upconversion Nanoparticles for Targeted Labelling of Bladder Cancer Cells
Source: Biomolecules. 2019 Dec 3;9(12):820. doi: 10.3390/biom9120820 (PMC6995529; doi:10.3390/biom9120820)
Supplement: Supplementary file 1 [file biomolecules-09-00820-s001.pdf]

biomolecules

MDPI

Supplementary Materials

Functionalized upconversion nanoparticles for targeted labelling of bladder cancer cells

Dmitry Polikarpov[1,2], Liuen Liang[1,2], Andrew Care[2], Anwar Sunna[2], Douglas Campbell[3], Bradley Walsh[3], Irina Balalaeva[5], Andrei Zvyagin[2,4,5], David Gillatt[1], Evgenii Guryev[5,*]

[1] Faculty of Medicine and Health Sciences, Macquarie University, NSW, Australia;
[2] Centre for Nanoscale Biophotonics, Macquarie University, NSW, Australia;
[3] Glytherix Ltd., Sydney NSW, Australia;
[4] The Institute of Molecular Medicine, I.M. Sechenov First Moscow State Medical University, 8-2 Trubetskaya str., Moscow 119991, Russia;
[5] The Institute of Biology and Biomedicine, Lobachevsky State University of Nizhny Novgorod, 23 Gagarin ave., Nizhny Novgorod 603950, Russia.
* Correspondence: eguryev@ibbm.unn.ru

Flow cytometry analysis of Glypican-1 expression by T24 and C3 cells

In order to demonstrate the ability of upconversion nanoconjugates coupled to MIL-38 antibodies to target Glypican-1 positive urothelial carcinoma, we used Glypican-1 high T24 and Glypican-1 low C3 (control) urothelial carcinoma cell lines kindly provided by Minomic International Ltd. Affinity of the monoclonal antibody MIL-38 towards urothelial carcinoma cells T24 was described previously[167] and was confirmed by the results of the flow cytometry analysis provided by Minomic International Ltd.

Flow cytometry was used to assess affinity of MIL-38 towards T24 and C3 cells. Firstly, T24 and C3 cells were incubated with antibodies MIL-38 and washed twice to remove unbound antibodies. Then, they were incubated with secondary antibodies, which were conjugated to a fluorescent label. These fluorescent secondary antibodies bound to MIL38 antibodies attached on the surface of cells. As a result, cells with MIL-38 on their surface were fluorescently labelled. T24 and C3 cells were subsequently analysed using a flow cytometer to assess their fluorescence intensity as a sign of the binding of MIL-38 to their surface. At least ten thousand cells were analysed in each group. Results of this analysis are presented in Figure S1, which shows the distribution of the fluorescence intensity of T24 and C3 cells. Flow cytometry analysis performed by Minomic International Ltd. demonstrated that the majority of T24 cells (Fig. S1, red histogram) had the stronger fluorescence than that of C3 cells. Minimal binding to C3 cells was also observed (Fig. S1, blue histogram). Secondary antibody alone was used as the control for this analysis (Fig. S1, black histogram).

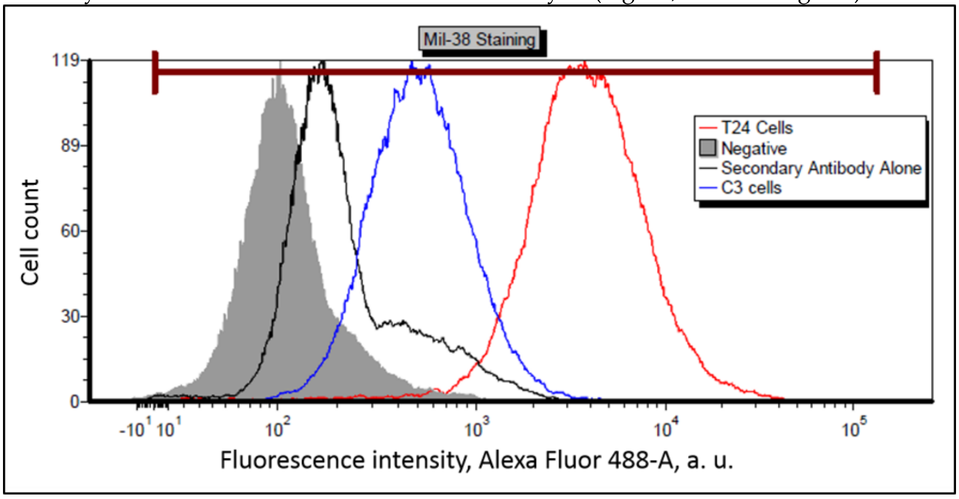

Figure S1. Flow cytometry analysis demonstrated the binding of MIL-38 antibody to T24 cells and minimal binding to C3 cells. Secondary antibody alone was used as a control. See text for details.
